# Recovery of pinch force sense after short-term fatigue

**DOI:** 10.1038/s41598-023-36476-8

**Published:** 2023-06-09

**Authors:** Lin Li, Yan-xia Li, Chong-long Zhang, Dong-hai Zhang

**Affiliations:** 1 Department of Physical Education, Renmin University of China, No. 59 Zhongguancun Street, Beijing, 100872 China; 2 College of Physical Education, Langfang Normal University, Langfang, Hebei China

**Keywords:** Sensory processing, Somatosensory system

## Abstract

The aim of this study was to identify the exact origin of force sense and identify whether it arises centrally or peripherally. The present study was designed to analyze the effects of short-term fatigue on pinch force sense and the duration of these effects. During the fatigue protocol, twenty (10 men and 10 women; M_age_ = 22.0 years old) young Chinese participants were asked to squeeze maximally until the pinch grip force decreased to 50% of its maximal due to fatigue. Participants were instructed to produce the target force (10% of maximal voluntary isometric contraction) using the same hand before and after fatigue (immediately, 10, 30, 60, 180, 300 s). The results showed significantly higher absolute error immediately after fatigue (1.22 ± 1.06 N) than before fatigue (0.68 ± 0.34 N), and 60 s (0.76 ± 0.69 N), 180 s (0.67 ± 0.42 N), and 300 s (0.75 ± 0.37 N) after fatigue (all P < 0.05) but with no effect on the variable error (*P *> 0.05). It was also revealed that there was a significant overestimate of the constant error values before (0.32 ± 0.61 N) and immediately after fatigue (0.80 ± 1.38 N, all *P *< 0.05), while no significant overestimation or underestimation exceeded 300 s after fatigue (*P *> 0.05). Our study results revealed that short-term fatigue resulted in a significant decrease in force sense accuracy, but it did not affect force sense consistently; however, force sense accuracy recovered to a certain extent within 10 s and 30 s, whereas it recovered fully within 60 s, and force sense directivity improvement exceeded 300 s after fatigue. The present study shows that the sense of tension (peripherally) is also an important factor affecting force sense. Our study supports the view that the periphery is part of the origin of force sense.

## Introduction

To attain precise and accurate muscle movement, proprioception sense, which is vital for individuals, is needed^1–3^. Proprioception (conscious) includes kinesthesia, force, and joint position sense^4–7^. The sense of force is the ability to accurately perceive the external or internal forces associated with each joint^8,9^. Many previous studies were designed to identify the exact origin of the force senses, and whether they are derived centrally or peripherally is of prime importance^10–12^. Studies have reported that there are two distinct sources of a muscle force sense, i.e., the sense of tension generated by afferent feedback from the muscle^13^ and the sense of effort generated centrally^14,15^. There is currently no consensus on which source of information is more important for force sense^10–12,16^.

Fatigue is defined as a disabling symptom in which physical and cognitive function is limited by interactions between performance fatigability and perceived fatigability^17–20^. Many psychophysical methods have been used to understand force sense and muscle fatigue, but “contralateral force matching” is the most widely used method^21^. In this method, participants are asked to generate a force by muscles contracting the reference along with external feedback, and the muscles of the contralateral matching limbs are used to match the subjective magnitude force without the assistance of feedback. When the muscle of the reference arm is fatigued, the effort that is needed to generate the required target force is far higher than the normal or regular use of the arm because the motor cortex excitability is reduced^15^. Hence, the participants’ indicator arm overshot the matching force while trying to match the efforts^15,22,23^. These studies^14,15,24–26^ support the view that sensations of muscle force arise from the sense of effort generated centrally. In addition to the above factors (the sense of effort), there may be other factors (the sense of tension) affecting fatigue. An ipsilateral force reproduction task is also a commonly used force sense test^11^. The indicator arm reproduces the previously generated reference force in the same hand in the ipsilateral force reproduction task, avoiding the interference of different forces between the tired (reference) arm and the untired opposite (indicator) arm and avoiding the interference of nerve transmission between the left and right hemispheres^27–30^. Therefore, to study whether factors other than force scaling have an impact on force sense during fatigue, the ipsilateral force reproduction task was adopted.

Numerous studies have investigated the effects of muscle fatigue on the ability to reproduce different force tasks^31–35^. Previous studies have reported that long-term fatigue deteriorates the ability of humans to make judgments about applied forces^32,36,37^. However, existing studies on the effect of short-term fatigue on force sense have reported inconsistent findings^37–39^. Pedersen et al*.* demonstrated deterioration of shoulder joint proprioception after shoulder muscle fatigue using a randomized controlled design that adopted a 30% peak torque^38^. However, in a different study utilizing the same value of the peak torque drop as the current study (50% maximal voluntary isometric contraction (MVIC)), the authors reported that shoulder muscle fatigue did not affect shoulder joint proprioception^39^. Spargoli et al*.* demonstrated similar results as in the current investigation^37^. Short-term fatigue does not affect the CNS (the sense of effort)^39–41^, but it will affect the periphery (the sense of tension)^42^. Therefore, to study whether the periphery has an impact on force sense, a short-term fatigue protocol was adopted in this study.

Existing studies on the relationship between sex and force reproduction errors have reported inconsistent findings. Bao et al. reported that sex and force reproduction were not related when comparing an observed palmar pinch force to an estimated force^43^, and other studies of grasp force matching errors did not find a sex issue^44^, while a previous study reported evident sex differences in a target force matching task, where men exhibited larger error values than women^45^. Emery et al. reported that there were no sex differences in shoulder or finger position accuracy before or after fatigue; however, there were sex differences in the perceived finger-target location and the temporal characteristics of the finger movement toward the target^46^. Furthermore, a recent analysis found that men were able to reproduce pinch forces more accurately and consistently than women^47^. To our knowledge, only one study has previously tested the effects of sex on force sense following muscle fatigue and has found significantly more force-matching errors in women than in men^32^.

Some studies have revealed that the short-term fatigue effects on force sense are not consistent. Furthermore, few studies have previously tested the effects of sex on force sense following muscle fatigue. Therefore, the purpose of this study was to induce the effects of short-term fatigue on the reproduction task of an ipsilateral force in both men and women, as well as to investigate the effects of a recovery period on these outcome measures. It was hypothesized that short-term fatigue would degrade pinch force sense, but it would return to pre-fatigue values quickly.

## Methods

### Participants

The participants in this study were 20 healthy people (an equal number of men and women) with an age of 22.0 ± 5.6 years, weight of 63.9 ± 14.4 kg, and height of 170.4 ± 9.6 cm, and all of them were right-handed. The dominant hand of the person was selected based on the hand they preferred for writing or doing work. The participants presented no neuromuscular disorders and were naive to the task. An informed written consent form was obtained from all of the participants after they were informed about the experimentation method. In addition, the participant signed informed consent to publish the image in an online open-access publication. The ethical review board of Renmin University of China (reference number 2021083) approved the present research/experiment. This study was performed in conformity with the principles of the Declaration of Helsinki.

### Apparatus

For strength and force reproduction testing, a pinch analyzer, which is a digital electronic force dynamometer (Kjyl Tech, Beijing, China), was used. The calibration and instrument validation settings were all checked before experimentation, and the device was calibrated as described by the manufacturer. For all of the tests, the pinch span was set at 2 cm, and a sampling frequency of 100 Hz was maintained. A protocol for the measurement of pinch force sense was developed based on the gathered data from the pinch analyzer.

### Protocol

**Figure 1:**
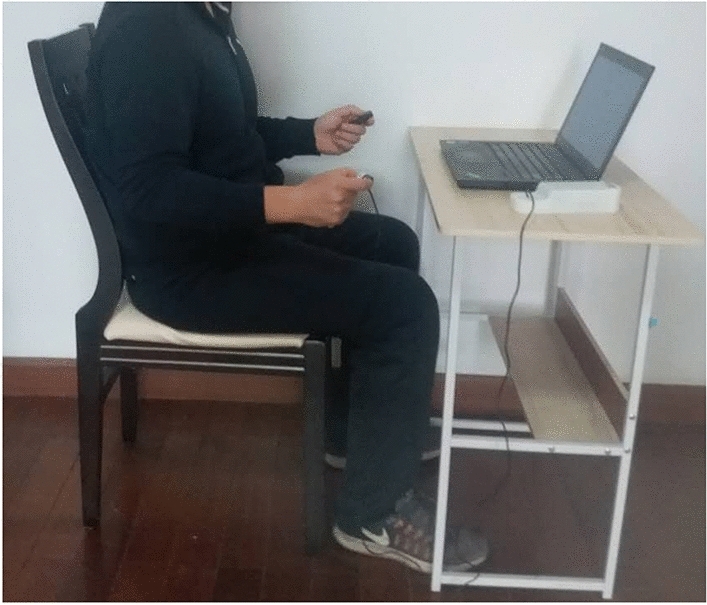
Standardized body and monitor positioning were used for the pinch force reproduction measurements.

All of the participants involved in the present study were forbidden from engaging in any unaccustomed upper limb exercises for one week before the experiment^48^. The study was also conducted in a quiet room to avoid or reduce any auditory distractions^49,50^. All of the participants were told to sit on a chair placed 60 cm in front of a computer table. The posture of the whole body was adopted as described by the guidelines of the American Society of Hand Therapists: neutral positions were followed for both the forearm and wrist, the elbow was flexed at 90°, and the vertical position was followed for the upper arm^51^. The isometric pinching tasks were performed by all of the participants by using a tip pinch because a pinch configuration is usually required for the task's precision^52^. In the tip pinch, all of the other fingers were fully flexed, and only the tip of the thumb to the index tip of the finger was involved in the movement (Fig. 1)^53^.

**Figure 2:**
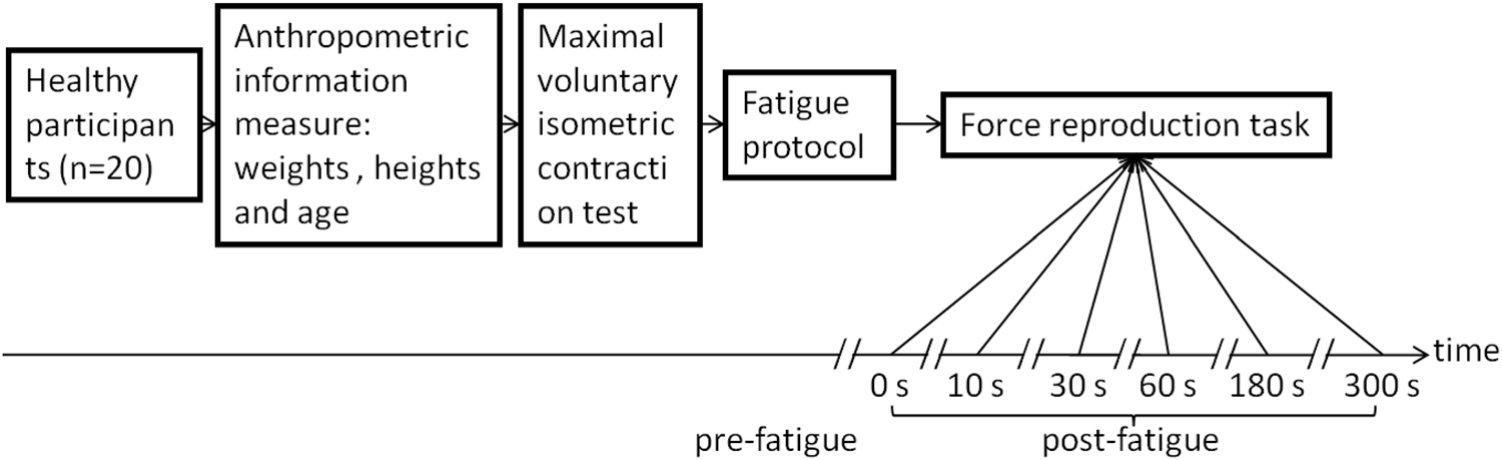
Flow chart of the trial carried out.

All of the experimental data were displayed on the computer monitor, and the participants were advised to maintain this posture configuration throughout the test. The force reproduction task and customized MVIC program test on the computer were used to analyze the data. The experimental protocols in this study included three steps as follows (Fig. 2):

#### Maximal voluntary isometric contraction test

All of the participants were advised to perform a warm-up exercise before testing. They were also told to perform a pinch grip and to apply a maximum pinch force on the dynamometer. The whole experiment was repeated twice, with the peak value of the two tests used to determine the pinch strength^43^. To reduce the fatigue impact on the experiment, all of the participants were given 2 min of rest time after every test.

#### Fatigue protocol

A marker was placed on the screen to indicate a value at 50% MVC and at 100% MVC to help motivate participants toward their maximal effort. During the fatigue protocol, the participant was requested to squeeze maximally until the pinch grip force decreased to 50% of its maximal due to fatigue^41,48,54^. The use of 50% MVC ensures a significant decline in function, allowing for the termination of the test at a time specific to each participant. The present investigation used this fatigue protocol to quantify the fatigue levels for each participant^39,41^. The time between the start of data collection and the time of task failure was denoted as the endurance time^55^. Verbal encouragement was also given by a single investigator, and attempts were made to maintain consistency between participants during all of the test procedures^41^.

#### Force reproduction task

**Figure 3:**
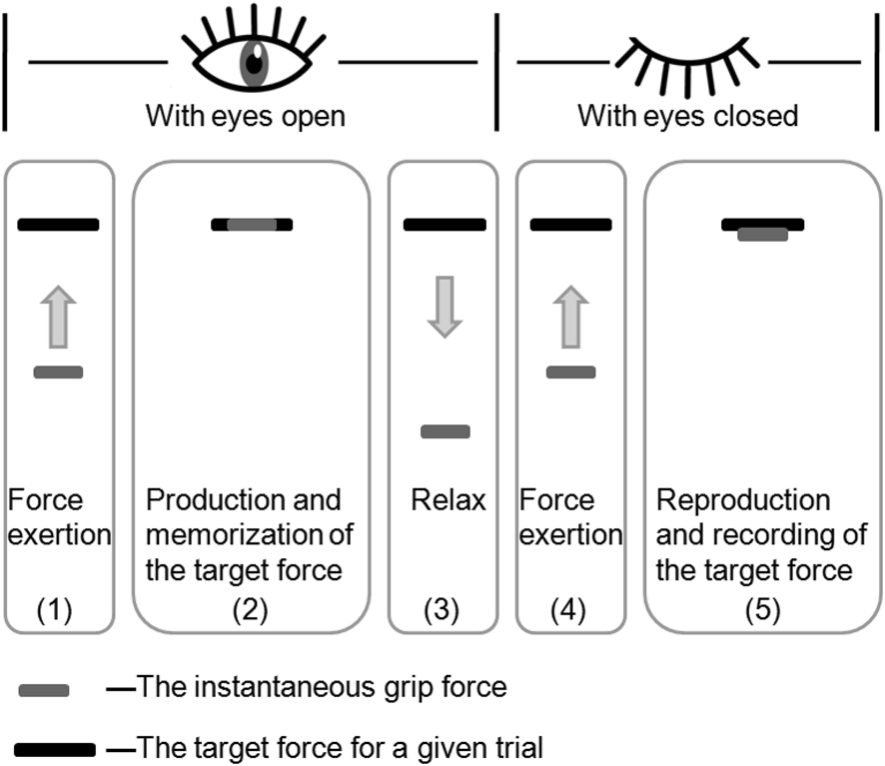
Schematic diagram of the computer display used to guide the participant to the target force during the experiment.

Force reproduction tasks were performed before and after fatigue (immediately, 10, 30, 60, 180, 300 s). All of the participants were given a visual demonstration first. The C +  + program was used in a given trial to assist all of the participants by showing them a black line that was a symbolizing target force, while a gray line on the screen was the representation of instantaneous pinch, as shown in Fig. 3. The participants were instructed to impart a target force (10% MVIC, T) using a pinch grip and to remember the force. Then, they closed their eyes and relaxed. Immediately, afterward, the participants regenerated the earlier force with the same fingers and without receiving visual feedback. They were also given a trigger to press when they believed they had applied the exact force required for the test, following which the computer recorded the exerted force (*R*_*i*_). All of the tasks were repeatedly performed until the participant was able to complete three sets of tasks (repetitions) in less than 10 s. Three repeated contractions were performed before and after fatigue (immediately, 10, 30, 60, 180, 300 s). Low force levels (10% MVIC) were chosen to minimize muscle fatigue^56,57^.

### Statistical Analyses

The dependent variables, i.e., absolute error (AE), constant error (CE), and variable error (VE), were selected to evaluate the errors in force sensing. Insight into the overall error is provided by AE, while insight into the error direction (i.e., under- or overestimation) is offered by CE, and how the error varies throughout a series of trials corresponds to VE. Thus, these variables reflect the precision of a participant in the test^58^. These parameters were calculated as follows:

1 $${\text{Absolute error }}\left( {AE} \right) = \frac{{\mathop \sum \nolimits_{i = 1}^{3} \left| { R_{i} - T} \right|}}{3},\left( {i = 1, 2, 3} \right),$$

2 $${\text{Constant error }}\left( {CE} \right) = \frac{{\mathop \sum \nolimits_{i = 1}^{3} \left( {R_{i} - T} \right)}}{3},\left( {i = 1, 2, 3} \right),$$

3 $${\text{Variable error }}\left( {VE} \right) = \sqrt {\frac{{\mathop \sum \nolimits_{i = 1}^{3} \left( {R_{i} - \overline{R}} \right)^{2} }}{3}} , \left( {i = 1, 2, 3} \right)$$

where *R*_*i*_ represents the reproductive force for the *i*th trial and *T* corresponds to the target force and $\overline{R}$ accounts for the mean across all three trials.

The Shapiro‒Wilk test confirmed the normality of the data. The MVIC differences between the sexes were determined by applying an independent t test. To compare the constant error to zero, a one-sample t test was performed to evaluate each point in time and to detect the trials in which excessively low or high forces were generated by the participants. To evaluate the time effects on absolute and variable error for pre-exercise and immediately, 10, 30, 60, 180, and 300 s post-exercise, a mixed-model analysis of variance was performed. The times and sex were considered within- and between-participant factors. Additional comparisons were also performed to check and verify the significant interactions and main effects. For multiple comparisons, Bonferroni-corrected post hoc comparisons were performed. The SPSS package (version 22.0) was used to analyze the experimental data. The data are represented as the means ± standard errors, and the significance threshold was considered to be *P *< 0.05.

## Results

### Maximal voluntary isometric contraction

There were significantly higher tip pinch forces in the men, i.e., 69.3 ± 16.2 N, than in the women (49.6 ± 9.4 N); t (18) = 3.32, *P *< 0.01). The MVIC of the women was 39.7% less than that of the men.

### Endurance time

The time required for the force to decrease up to 50% MVIC is known as the endurance time. There was no significant difference in endurance time between men (26.7 ± 10.7 s) and women (30.6 ± 15.1 s; t (18) = -0.66, *P *> 0.05).

### Absolute, variable, and constant error

**Figure 4:**
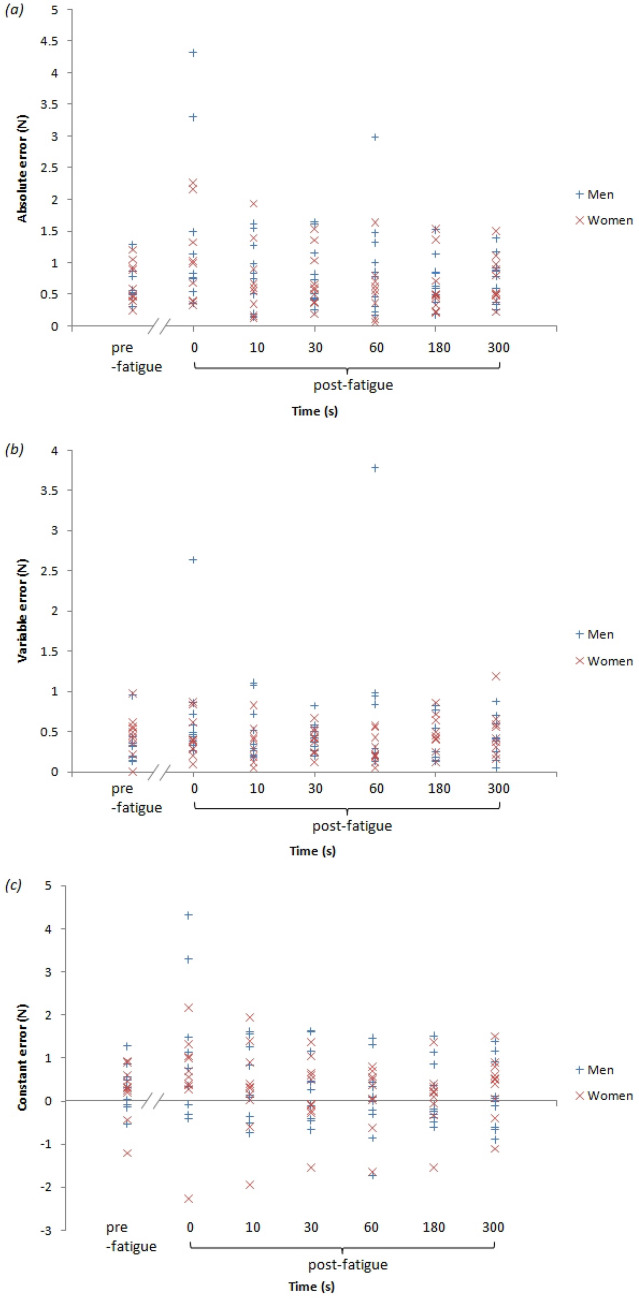
The individual accuracy, direction, and precision values were assessed based upon (**a**) absolute error, (**b**) constant error, and (**c**) variable error as a function of participant sex and time.

**Figure 5:**
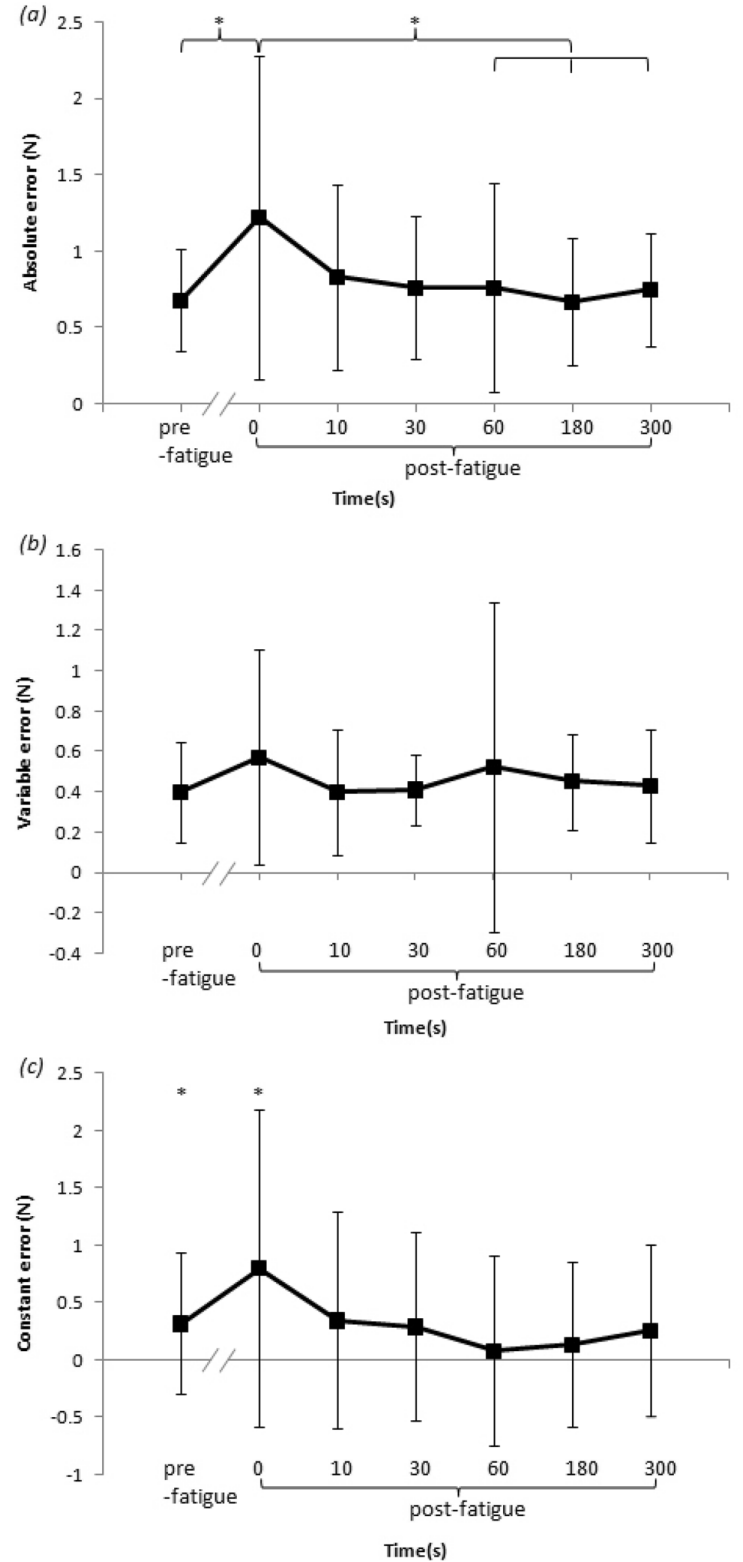
(**a**) Absolute error was used to assess the overall error in force reproduction based on time. (**b**) The variable error was used to assess the variability in error among the trials as a metric for individual performance based on times. (**c**) Constant error, the force of the reproduction directionality of error, as a function of time ( *P *< 0.05).

The individual data of the absolute, constant, and variable error are presented in Fig. 4. Mixed-model ANOVA was used to compute individual absolute and variable errors. The results revealed no significant interaction between time and sex for absolute error (F (6, 108) = 0.66, *P *> 0.05) or variable error (F(6, 108) = 1.65, *P *> 0.05). Sex did not have a significant main effect on absolute error (men: 0.87 ± 0.71 N, women: 0.75 ± 0.51 N, (F (1, 18) = 0.36, *P *> 0.05) or variable error (men: 0.51 ± 0.54 N, women: 0.40 ± 0.24 N, F(1, 18) = 1.03, *P *> 0.05). There was a significant main effect of the times for absolute error (F (6, 108) = 3.39, *P *< 0.01), while a nonsignificant effect was obtained by variable error (F (6, 108) = 0.64, *P *> 0.05). The results revealed a significantly higher absolute error immediately after fatigue (1.22 ± 1.06 N) than before fatigue (0.68 ± 0.34 N) and 60 s (0.76 ± 0.69 N) and 180 s (0.67 ± 0.42 N), 300 s (0.75 ± 0.37 N) after fatigue (all *P *< 0.05). This indicates that force sense fully recovers within the 60 s after fatigue. Additionally, no significantly different in absolute error was identified between before fatigue and post 10 s (0.83 ± 0.60 N, *P *= 0.252), 30 s (0.76 ± 0.47 N, *P *= 0.363). Furthermore, the absolute error immediately after fatigue was higher than at 10 s (*P *= 0.061), and 30 s (*P *= 0.062) after fatigue, indicating that even though there was no statistical significance, force sense will recover to a certain extent within 10 s and 30 s (Fig. 5a,b. Significantly higher constant error values were detected before fatigue (0.32 ± 0.61 N, t (19) = 2.33, *P *< 0.05) and immediately after fatigue (0.80 ± 1.38 N, t (19) = 2.60, *P *< 0.05), while no significantly lower or higher constant error values were observed at any other times (t (19) = 0.46–1.64, all *P *> 0.05; Fig. 5c).

## Discussion

Our study results revealed that short-term fatigue resulted in a significant increase in absolute error but had no effect on variable error immediately after fatigue; however, force sense accuracy recovered to a certain extent within 10 s and 30 s, whereas it recovered fully within 60 s. Our study results also revealed a significant overestimate of the constant error values before and immediately after fatigue, while no significant overestimation or underestimation exceeded 300 s after fatigue. Additionally, sex did not have a significant effect on the recovery of force sense after fatigue.

### The immediate effects of fatigue on force sense

The results revealed a significantly higher absolute error immediately after fatigue than before fatigue and 60, 180 and 300 s after fatigue but no effect on the variable error. Significantly higher constant error values were detected before and immediately after fatigue. Studies on the effect of short-term fatigue on proprioception have shown that fatigue involves several peripheral changes, including an altered metabolic state, muscle activation patterns, and muscle spindle discharge^59,60^. The high concentrations of metabolites and inflammatory products produced during muscular contraction (for example, lactic acid, arachidonic acid, bradykinin, potassium, and prostaglandin E2) cause an increase in the muscle spindle discharge rate, greater alpha-gamma coactivation, and nociceptor activation in fatigued muscle^61,62^. Information regarding muscle forces and interaction forces is detected by the Golgi tendon organs (GTOs) in the muscles^63–66^. Therefore, after a series of fatigue tasks, there are significantly higher absolute errors due to the GTO discharge being affected by muscle fatigue. Additionally, there is the influence of pain, muscle stiffness, muscle coordination deterioration, etc., caused by muscle fatigue. Some part of the apparent reduction in proprioception may be related to the pain that diverts attention from proprioception^56,67,68^. Evidence in favor of this idea is that saline-induced pain in unexercised muscles gave rise to force reproduction errors of a size comparable to those observed after fatigue exercise^69,70^. In the presence of pain, proprioception can be disturbed due to altered reflex activity and sensitivity of the gamma-muscle spindle system^71^ via activation of chemosensitive type III and IV afferents (nociceptors)^72–75^. Moreover, pain can influence body perception at the central level^76,77^, including reorganization of the somatosensory cortex^78^. Thus, pain can negatively influence proprioception at both the peripheral and central levels of the nervous system. Another possible source of the force reproduction errors considered here was the increased muscle stiffness. After fatigue exercise, the increased muscle stiffness may alter the responses of force sense receptors such as the GTOs and thus affect the force sense at the finger^56,79^. Another adjustment that may reduce force sense during muscle fatigue is a change in muscle coordination patterns^55,59^ and a decrease in executive attention^6^. Indeed, several peripheral mechanisms may act simultaneously.

An ipsilateral force reproduction task was adopted in this study to avoid the interference of nerve transmission between the left and right hemispheres^27,28^. Additionally, the fatigue caused by the short-duration, high-intensity protocol affected only the periphery in this study. The ipsilateral force reproduction task and fatigue protocol in this study did not affect the sense of effort, but they did affect the sense of tension. The effects of fatigue on the absolute error in this study support the view that the sense of tension is also an important factor affecting force sense.

The results also revealed significantly higher constant error values before and immediately after fatigue. Many previous studies revealed participants’ tendency to exert excessive force at lower target force levels and insufficient force at high force levels^80–84^. The 10% MVIC (lower force level) was used as the target force; therefore, it was overestimated before and immediately after fatigue in this study.

### Recovery of force sense after fatigue

Although many studies have found that exercise-induced muscular fatigue immediately affects proprioception^36,39,62,85,86^, few studies have investigated the recovery of proprioception after fatigue^54,87^. The recovery time necessary for absolute error (< 60 s) is shorter than for constant error (> 300 s). No significant overestimation or underestimation was detected at 30, 60, 180, and 300 s after fatigue. The endurance time depends on the fatigue type protocol. Overall, this study is consistent with a previous finding^88^ of no significant difference in endurance time between men and women or endurance time independent of muscle strength. The average endurance time value was 26.7 ± 10.7 s for men and 30.6 ± 15.1 s for women. These obtained values are similar to those reported in other published works (26.0 and 35.4 s)^35^. The endurance time in this study was short, so it can be quickly recovered from.

Some studies have suggested that fatigue affects the force sense^62,85^, but others have found the opposite^36,39,86^. It was postulated that the contradictory results of different studies may be a result of the amount of time that elapsed between the fatigue protocol and the force sense acuity test and the variables for force sense error evaluation (absolute error, constant error, or variable error). There are many variations in fatigue protocols among investigations. The results from previous studies indicated that the intensity, mode, and duration of exercise and the muscle group involved might all play a vital role in the resulting state of fatigue and recovery following fatigue. Participants experiencing a greater amount of muscle fatigue took longer to recover following the fatigue protocol^36,62^. Additionally, a longer recovery period was found following tasks with low intensity and long duration versus high intensity with short duration^89^. The shorter time needed for the recovery of AE may be a reflection of this study’s fatigue protocol and the small finger muscles involved. The current study used continuous maximal contractions to induce finger muscle fatigue. This type of exercise-induced fatigue causes the energy in the muscle tissue to be rapidly depleted. Thus, there may have been an insufficient supply of bioenergy for the muscle fibers to subsequently execute movements. However, after a few moments of rest, the biochemical metabolism restored the bioenergy reserves. Hence, this type of exercise-induced fatigue rapidly led to muscle fatigue yet permitted rapid recovery to pre-fatigue levels.

Some studies have demonstrated that a shorter time needed for the recovery of absolute error may have an insignificant effect on force sense^37,39^ when the force sense tests are performed at an interval of more than 10 s after the fatigue protocol. The results of this investigation revealed no significant difference in absolute error between before fatigue and at 10 s and 30 s after the fatigue, concluding that forces sense also recovers to a certain extent within 10 s and 30 s. Therefore, the precise methods used in the present study not only clarify the conflicting findings from previous studies^37–39^ but also confirm that short-term fatigue decreases force sense accuracy (absolute error) and directivity (constant error) by affecting the GTOs but also that force sense consistency (variable error) may not vary as long as the muscle is in a fatigued condition.

Significantly higher constant error values were detected before and immediately after fatigue, while no significantly lower or higher constant error values were observed 10, 30, 60, 180, and 300 s after fatigue. Another important point in the present study was the force sense directivity improvement after fatigue, supporting the results of previous studies. Romero-Franco et al. reported proprioceptive improvement twenty-four hours after “lactic exercise” when the joint position sense improved concerning the baseline^90^. Although Kennedy et al.^91^ reported a postural control impairment following a mild fatigue protocol, athletes improved their postural control ten minutes later compared to baseline. Since these postural control improvements remained after muscle strength recovery, the authors suggest “adjustments centrally mediated protective response as opposed to a peripherally induced limitation to performance”. These findings are in line with those of Brown et al*.*^92^, who stated that prolonged proprioceptive stimulation by practicing physical activity implies medium- and long-term adaptations that improve the motor control of athletes.

The results also showed that sex did not have a significant effect on pinch force sense before and after fatigue. Few studies have investigated the muscular fatigue-affected force sense between sexes^32^. Existing studies on the relationship between sex and force reproduction errors have reported inconsistent findings^32,43,45^. Furthermore, this study on grip force sense revealed significantly lower absolute error values for higher force levels of 90–130 N among women than men; however, significant sex differences for lower force levels of 10–80 N were not noted^93^. The 10% MVIC (lower force level) was used as the target force; therefore, sex did not have a significant effect on pinch force sense before fatigue in our study.

Although there have been many studies on the relationship between sex and force sense, no studies have investigated the recovery of force sense between sexes. The recovery of the force sense depends on the fatigue type protocol. The participants were asked to squeeze maximally until the pinch force decreased to 50% of its maximal due to fatigue in our fatigue protocol. The present investigation used this fatigue protocol in an attempt to quantify fatigue levels between sexes. Overall, our study showed no significant difference in endurance time between men and women or in endurance time independent of muscle strength. Therefore, sex did not have a significant effect on the recovery of force sense after fatigue in our study. However, the strength required to perform manual tasks at work, such as turning screws, is absolute. An absolute force of the same magnitude may be more demanding among women than among men and may lead to greater fatigue in women. Fatigue is caused by absolute forces that may have different effects by sex. Therefore, further research on the effects of absolute force-induced fatigue on sex is needed.

### Limitations

Our study has some limitations. For example, the small sample size is insufficient to represent the population. Only healthy young adults, with a mean age of 22.0 years, were enrolled in this study. Therefore, the conclusions in the study may be valid only for the assessment of pinch force sense in healthy individuals of similar age. Thus, additional studies are needed to examine these relationships among other demographics. In addition, the learning effect was not studied in this article. This study measured the pinch force as a single value. However, the force from the two fingers (thumb and index finger) was asymmetrical^94^. Separate measurement of force sense from each finger and evaluation of the differences between two fingers in terms of the effect of fatigue on motor control accuracy are planned for further studies.

## Conclusion

The present study revealed that short-term fatigue resulted in a significant decrease in force sense accuracy (absolute error) but did not affect force sense consistently (variable error); however, force sense accuracy recovered to a certain extent within 10 s and 30 s, whereas it recovered fully within 60 s, and force sense directivity (constant error) improvement exceeded 300 s after fatigue. Additionally, there was no sex difference in the effect of fatigue on force sense. The effects of short-term fatigue on absolute error tested by the ipsilateral force reproduction task in our study support the view that the sense of tension is an important factor affecting force sense. Knowledge of these mechanisms will enhance our understanding of the origin of the force senses and the fatigability and recovery capability of force sense, assisting in the application of better exercise programs in both clinical and training settings and thereby helping to enhance muscle performance and reduce the risk of injury.

## Data Availability

The datasets used and/or analyzed during the current study are available from the corresponding author on reasonable request.
